# Socioeconomic status and adiposity in childhood cancer survivors: A cross-sectional retrospective study

**DOI:** 10.1371/journal.pone.0298068

**Published:** 2024-02-16

**Authors:** Lucie Štrublová, Tomáš Kepák, Daniela Kuruczová, Filip Zlámal, Marta Holíková, Kateřina Kepáková, Jaroslav Štěrba, Julie Bienertová-Vašků

**Affiliations:** 1 Department of Pathological Physiology, Faculty of Medicine, Masaryk University, Brno, Czech Republic; 2 International Clinical Research Center, St. Anne’s University Hospital Brno, Brno, Czech Republic; 3 Department of Paediatric Oncology, University Hospital Brno, Masaryk University, Brno, Czech Republic; 4 Department of Food Technology, Mendel University, Brno, Czech Republic; 5 Department of Physical Activities and Health Sciences, Faculty of Sports Studies, Masaryk University, Brno, Czech Republic; Shahid Beheshti University of Medical Sciences, ISLAMIC REPUBLIC OF IRAN

## Abstract

This is a retrospective cross-sectional study examining the association between unemployment, cancer type, treatment and total body fat percentage of childhood cancer survivors recruited at St. Anne’s University Hospital in Brno, Czech Republic. A total of 55 survivors aged 18–49 who were in remission of cancer and fulfilled the criteria for body composition measurements by the BIA and completed questionnaires investigating their socioeconomic status, employment status, and history. There was a significant relationship between the employment status and central nervous system-directed treatment (c^2^(1) = 7.53, p = 0.006, Cramér’s V = 0.38) and between the type of cancer and employment status (c^2^(3) = 7.83, p = 0.049, Cramér’s V = 0.38), the highest unemployment rate was recorded for brain and spine survivors (72.7%) compared to survivors with other diagnosis (35.7%) (uLR(1) = 4.91, p = 0.027; OR = 4.80, 95% CI:1.10–20.86, p = 0.036); these survivors did not have a significantly different body fat percentage compared to survivors with other diagnoses (t(53) = 1.29, p = 0.202, Cohen’s d = 0.41) Interestingly, the survivors reporting having a partner also had a significantly higher percentage of body fat (t(53) = 2.90, p = 0.005, Cohen’s d = 0.81). A linear regression model was used to model the percentage of body fat in relation to a set of selected variables and the we observed a significant effect of sex (female vs male: b = 6.37, 95% CI: 1.82–10.93, p = 0.007), partnership status (yes vs no: b = 5.65, 95% CI: 0.67–10.62, p = 0.027) and category of diagnosis (Brain and spinal column tumors vs Other solid tumors: b = 12.40, 95% CI: 0.59–24.21, p = 0.040; Brain and spinal column tumors vs Lymphoma: b = 14.02, 95% CI: 2.06–25.97, p = 0.023). Employment status and risk of adiposity in childhood cancer survivors depends on the type of treatment and diagnosis group, which may significantly impact their lifestyle and overall quality of life after treatment.

**Trial registration**: This study was registered on July 29, 2022, at ClinicalTrials.gov (NCT05481229).

## Introduction

Almost 80% of children survive childhood and adolescent cancers in Europe [1]. Although late effects of treatment can affect many survivors, not all survivors experience severe late effects. For this reason, it is important to know the nature of possible health complications after treatment and the factors behind the development of these complications [2]. The most common problems in this patient population include psychosocial problems, including cognitive dysfunction, endocrinopathies including growth disorders, infertility, hypopituitarism, hypothyroidism, metabolic syndrome, and nutritional disorders. Childhood cancer survivors are at greater risk of becoming overweight and obese, which increases their already high risk of cardiac morbidity and mortality [3]. However, this is only true for some types of diagnoses. According to available studies [4–6], survivors after acute lymphoblastic leukaemia (ALL) and brain and CNS tumours are most at risk of increased adiposity. The excess adipose tissue accompanying obesity alters the release of adipokines, proteins produced by adipocytes. Adipokines such as leptin and adiponectin affect cellular insulin sensitivity and vascular function, increasing the risk of metabolic syndrome and associated cardiometabolic complications [7–9]. Risk factors for adiposity are reduced physical activity, sedentary behaviour, and cranial irradiation with identified or presumed hormonal dysregulation. Cranial irradiation at higher doses, such as those used to treat brain tumours, is the most common treatment associated with obesity [10].

The moderate doses of cranial irradiation (1.200–2.400 Gy) used to treat childhood ALL have been associated with obesity, including central, liver, and visceral obesity [11, 12]. Mechanisms by which cranial irradiation may contribute to obesity include damage to the hypothalamus, including disruption of the growth hormone, thyroid, and gonadal function, as well as changes in sensitivity to leptin, ghrelin, and insulin [13]. A specific type of cancer treatment with multiple undesirable effects on the later life of childhood cancer survivors (CCS) is treatment targeting the central nervous system (CNS). The available studies show that this treatment [14, 15] can cause long-term academic, social, and emotional difficulties for children cancer survivors. Indeed, neurosurgery, cranial radiation, and intrathecal chemotherapies have all been associated with neurocognitive difficulties. It is important to look at both diagnosis and treatment type when determining the risk for long-term difficulties [14]. Other contributors can be family risk and socioeconomic factors [16].

Socioeconomic status (SES), a multi-dimensional construct encompassing economic resources, power, and social standing, has been associated with multiple health outcomes [17]. Further studies [18–21] suggest that socioeconomic status impacts health outcomes in the CCS cohort. Socio-economic outcomes for survivors are also lower compared to healthy siblings, and fewer survivors graduate from college and obtain full-time employment [22]. Many studies show that unemployment negatively affects various aspects of health. The cumulative length of unemployment is correlated with deteriorated health and health behaviour. In Germany, a study based on the German Childhood Cancer Registry revealed a % unemployment rate of 21% among survivors compared to 16% in controls. Despite this, the probability of survivor unemployment remained lower in Europe than, for example, in the US and Canada [21]. Survivors of central nervous system tumours were up to five times more likely to be unemployed than the control group. Women were more often unemployed than men [21]. Previous reviews showed that neuro-cognitive impairments related to intelligence, memory, and executive functioning (essential for staying in the workforce) are common in CNS tumour survivors [13, 23].

The cumulative duration of unemployment correlates with poorer health status and health behaviours and lower physical activity [15, 22]. Unemployment can lead to higher levels of stress and a subsequent increase in stress hormones (e.g. cortisol, catecholamines, glucagon and growth hormone). These processes can ultimately alter fat storage and increase the amount of visceral fat [16, 19, 24, 25].

## Materials and methods

### Study design

This paper presents data from the NUTRITION CCS research project, a retrospective cross-sectional study of a sample of 73 survivors of childhood cancer who received follow-up medical care at the outpatient Late Effects Clinic at St. Anne’s University Hospital in Brno, Czech Republic. This study evaluates the relationship between body composition, selected indicators of socioeconomic status, cancer type and treatment. All procedures involving human participants were in accordance with the ethical standards of St. Anne’s University Hospital in Brno. The study was approved by the Local Ethics Committee and approval for this research was granted under registration number IIT/2017/35.

#### Study setting

The outpatient Late Effect Clinic opened in 2016 at St. Anne’s Hospital in Brno, Czech Republic, and enrolled aged 18 years and older. Prior to enrolment in the study each participant signed an informed consent form to participate in the study. Annually, 350 survivors visit this clinic, for a total of 450 visits yearly. The average age of survivors is 30 years. The survivors were recruited in person during their routine visits to the survivorship clinic. A total of 73 individuals were approached and 71 of them provided informed consent to participate in the study. The recruitment for this study began on April 25, 2018, and the last survivor for data evaluation for this manuscript was recruited on June 5, 2019. A present researcher completed the study questionnaire with survivors during their clinic visit. The questionnaire included two parts. The attending physician and researcher completed the first part. This section included questions about anthropometric data, the survivor’s personal history, including the disease’s family history, and questions about the type of cancer and length of treatment. The survivor then completed the second part. This section included questions on socioeconomic status, such as ethnicity, marital status, housing, educational attainment, partner, occupation, and salary level. There were also questions about quitting, loneliness, family history, pharmacological history, injuries, allergies, surgeries, reproductive history, gynaecological history, and length of time outdoors. The exact wording of the questions analysed for this study is provided in this article’s "Socioeconomic Status" section. Survivors were measured by bioelectrical impedance analysis (BIA) at the time of recruitment into the study, and their height, weight, waist, and hip circumference were recorded.

#### Participants

The survivors were 18–49 years old (mean age 24.98 years) and had been diagnosed with childhood cancer in the period 1983–2011. For the study, we recruited a consecutive series of survivors who came to the outpatient clinic for medical examination and met the following criteria: a) had an appointment at the outpatient clinic between April 2018 and June 2019; b) were treated with chemotherapy and/or radiotherapy for cancer diagnosed at the age of 0–18 years; c) were aged 18 years or older; d) were in complete remission of primary cancer disease; e) were meeting the BIA measurement criteria; f) have signed an informed consent approving the use of their health data for scientific and research purposes of this study.

#### Anthropometric characteristics

The total body fat percentage was measured using the InBody model 370. All survivors were instructed on fluid and food intake before body composition measurements. Instructions are given on the official website of the manufacturer of this device [26]. The patients with a missing upper or lower limb, pregnant women and the patients with a pacemaker were excluded from the study.

For the analysis, the body fat percentage was categorized according to the Obesity Medicine Association (OMA). According to this classification, an increased amount of fat between 30–34% in women and 25–29% in men is considered risky and is considered a pre-obesity status. Body fat content values >35% in women and >30% in men were then defined as obesity. The body fat percentage was used as a continuous variable in structural equation modeling with adjustment for sex. The weight was measured to the nearest 0.1 kg on an electronic scale and confirmed by weight measurement from the In Body 370 device. The height was measured using a stadiometer. Based on the height and weight obtained, the body mass index was calculated by dividing weight in kilograms by height in centimetres squared. Finally, the waist circumference was measured using a measuring tape in the horizontal plane at a point marked just above the right subcostal bone on the mid-axillary line [27], with minimal breathing, and classified according to WHO [28]. Height and circumference were recorded to the nearest 0.1 cm.

#### Socioeconomic status

The socioeconomic status was evaluated from the questionnaire that included the following questions “What is your highest level of education? (a) It is not known; (b) Elementary School; (c) Vocational school without a school-leaving certificate exam; (d) Vocational school with a school-leaving certificate exam; (e) Grammar school; (f) Higher vocational school; (g) University; (h) Postgraduate study”. The responses were categorized into: It is not known (a), Elementary School (b, c), High school (d, e, f), and University (g, h).

The questionnaire also included the following questions: “Do you have a steady partner? (a) Yes; (b) No”. “Do you have a job? (a) Yes; (b) No”. “What is your current income? (a) Very low (gross earnings of <5000 CZK per month); (b) Low (gross earnings of 5000–14999 CZK per month); (c) Average (gross earnings of 14999–24999 CZK per month); (d) Higher than average; (e) High (gross earnings of > 35000 CZK per month)”. These responses have been categorized into Below average (a, b), Average (c), and above average (d, e).

#### Type of treatment

Types of treatment included the CNS-directed or non-CNS-directed treatment received throughout the survivor’s treatment. CNS-directed treatment included one or a combination of the following: (a) intrathecal chemotherapy and (b) cranial irradiation (including whole-body radiation) or (c) neurosurgical interventions (e.g., partial or complete tumour resection). These data were used to create the dichotomous variable "type of treatment" to reflect receipt of any treatment vs. no treatment directed to the CNS.

### Analytical sample

The analysis included 55 survivors who had completed the anthropometric data, data on oncology treatment, and socioeconomic data. The study included 73 survivors, 10 of whom did not complete the questionnaire and 8 did not meet the criteria for BIA measurement. Two survivors had missing data on the education and employment status questions.

#### Data analysis

The data analysis was performed using statistical software R version 4.0.3 [29]. First, descriptive analysis was conducted, and basic relationships between variables were assessed. The data analysis needs to respect the character of the was performed in respect to the character of the obtained data. In our case, the variables: sex, diagnosis, education, income, relationship status, employment status, CNS-directed treatment and type of cancer were considered as categorical variables whereas height, weight, BMI, age, age at diagnosis, body fat percentage, waist, hips and event free survival (EFS) were considered as continuous variables, distributions of categorical variables were characterized by their absolute and relative frequencies. In the case of continuous variables, the mean and standard deviation were used. The relationship between categorical variables was assessed using Pearson chi-square test (with Cramer’s contingency coefficient as effect size) and odds ratio was calculated (if possible). Several statistical tests were used to identify differences in distributions of continuous variables between levels of selected categorical variables. For this purpose, parametric (t-test with Cohen’s d as effect size, Welch test with Cohen’s d as effect size, ANOVA with eta-squared as effect size) as well as non-parametric (Mann-Whitney test with r coefficient as effect size, Kruskal-Wallis test with eta-squared as effect size) tests were used.

Finally, linear regression model for body fat percentage as dependent variable with diagnosis, CNS-directed treatment, sex, partnership status, unemployment status, event free survival and age as independent variables was created. Subsequently, assumptions of linear regression model were checked (normality, homoscedasticity, independence of residuals).

## Results

### Survivor characteristics

Out of the total of 55 childhood cancer survivors who were included in the analysis, there were 24 women and 31 men. Further information on the demographic/anthropometric characteristics of the sample can be found in Table 1. During exploratory analysis, strong correlations were found between height, weight, waist, hips, and BMI (height vs weight: r = 0.65, p<0.001; height vs waist: r = 0.45, p<0.001; height vs hips: r = 0.40, p = 0.002; height vs BMI: r = 0.25, p = 0.057; weight vs waist: r = 0.80, p<0.001; weight vs hips: r = 0.86, p<0.001; weight vs BMI: r = 0.87, p<0.001; waist vs hips: r = 0.82, p<0.001; waist vs BMI: r = 0.77, p<0.001; hips vs BMI: r = 0.84, p<0.001). Body fat percentage was moderately positively correlated with waist (r = 0.36, p = 0.007), hip measurements (r = 0.44, p<0.001) and BMI (r = 0.51, p<0.001) and moderately negatively correlated with height (r = -0.33, p = 0.011), and also not significantly correlated with weight (r = 0.19, p = 0.151). The expected differences between sexes in height, weight, waist, and body fat percentage were also observed. When evaluating body fat percentage measurements according to the Obesity Medicine Association (OMA) [30], the distribution of fat percentage into categories. It was found that 9.1% CCS were in category of pre-obesity and 18.1% were obese. The socioeconomic characteristics of the research sample are also presented in Table 1B. An expected positive association was found between education, employment status, and income. The variable income was excluded from further analysis due to many missing values and a strong positive association with education and employment status.

**Table 1:** A) Anthropometric characteristics, age, body fat and socioeconomic status of the sample by sex. Mean (standard deviation) values are presented. B) Distribution of selected categorical variables (body fat percentage category, education, income, partnership and employment status).

**Table 1A**								
**Variable**		**Male (N = 31)**	**Female (N = 24)**	**Total (N = 55)**	**Test**	**Test statistic**	**p**	**Effect size**
Height (cm)		175.9 (10.09)	167.3 (6.88)	172.2 (9.75)	Welch	3.75	<0.001	0.88 (large)
Weight (kg)		71.8 (16.20)	60.1 (9.35)	66.7 (14.74)	t-test	3.14	0.003	1.00 (large)
BMI (kg/m^2^)		23.1 (4.23)	21.4 (2.85)	22.3 (3.75)	t-test	1.62	0.111	0.45 (small)
Body fat (%)		19.9 (8.80)	27.1 (7.00)	23.1 (8.77)	t-test	-3.29	0.002	0.91 (large)
Waist (cm)		84.4 (11.99)	75.1 (10.24)	80.3 (12.08)	t-test	3.01	0.004	0.83 (large)
Hips (cm)		91.0 (9.72)	89.6 (7.60)	90.4 (8.81)	t-test	0.60	0.553	0.16 (negligable)
Age (y)		24.99 (5.52)	25.06 (5.60)	24.98 (5.41)	Mann-Whitney	-0.81	0.425	0.11 (small)
Variable	Category	N (%)						
Body fat	Essential fat	6 (10.9)						
	Fitness	9 (16.4)						
	Athlete	9 (16.4)						
	Acceptable	16 (29.1)						
	Pre-obesity	5 (9.1)						
	Obesity	10 (18.1)						
Education	Elementary	17(30.9)						
	High school	27(49.1)						
	University	11(20.0)						
	Missing	0(0.0)						
Income	Below average	17(30.9)						
	Average	13(23.6)						
	Above average	9(16.4)						
	Unknown	16(29.1)						
Relation-ship status	In relationship	23(41.8)						
	Single	32(58.2)						
	Missing	0 (0.0)						
Employment status	Employed	30 (54.5)						
	Unemployed	23 (41.8)						
	Missing	2 (3.6)						

Effect size: t-test–Cohen’s d, Welch test–Cohen’s d, Mann-Whitney test—r

Table 2 shows the results of EFS, age at diagnoses, body fat by CNS-directed treatment and type of cancer.

**Table 2:** Cancer diagnosis and related characteristics. The mean (standard deviation) is presented for EFS, age at diagnosis, and body fat percentage.

Variable		Analytic sample				
		(N = 55)				
		N	%	EFS (years)	Age at diagnosis (years)	Body fat (%)
CNS-directed treatment	Yes	19	34.5	12.1 (5.2)	7.6 (3.7)	24.5 (10.0)
	No	36	65.5	11.6 (6.8)	12.2 (4.4)	22.3 (8.1)
test				t-test	MW	t-test
Test statistic				-0.27	3.26	-0.89
p				0.785	0.001	0.376
Effect size				-0.08 (negligable)	0.44 (moderate)	-0.25 (small)
Type of cancer	Brain and spinal column tumours	11	20.0	13.6 (4.4)	7.8 (4.7)	26.1 (10.1)
	Leukaemia	8	14.5	10.9 (4.4)	6.9 (1.6)^a,b^	22.3 (7.3)
	Lymphoma	20	36.4	9.8 (7.6)	14.0 (3.1)^a^	21.4 (7.6)
	Other solid tumours	16	29.1	13.4 (6.0)	10.1 (4.8)^b^	23.3 (10.0)
test				ANOVA	Kruskall-Wallis	ANOVA
Test statistic				1.38	15.93	0.67
p				0.259	0.001	0.574
Effect size				0.08 (small)	0.25 (large)	0.04 (small)

Effect sizes: t-test–Cohen’s d, Mann-Whitney test–r, ANOVA—eta-squared, Kruskal-Wallis–eta-squared. Groups with the same superscript significantly differ.

### Cancer diagnosis, CNS-directed treatment, and socioeconomic status

Using Pearson’s Chi-squared test, a significant relationship was found between the type of cancer and employment status (c^2^(3) = 7.83, p = 0.049, Cramér’s V = 0.38) and between employment status and CNS-directed treatment (c^2^(1) = 7.53, p = 0.006, Cramér’s V = 0.38). Unemployment rates also varied across diagnosis groups. The highest unemployment rate was in the CCS group diagnosed with “Brain and spinal tumours” (72.7%), compared to survivors with other diagnosis (35.7%) (uLR(1) = 4.91, p = 0.027; OR = 4.80, 95% CI:1.10–20.86, p = 0.036). Other relationships came out statistically insignificant.

### Cancer diagnosis and body fat percentage

A linear regression model was fitted to explore the association between the body fat percentage and cancer diagnosis. To control for other variables associated with body fat percentage or diagnosis, the following covariates were included in the model: sex, relationship status, employment status, CNS-directed treatment, and EFS. The results for the regression model are presented in Table 3. The regression model showed that survivors reporting having a partner also had a significantly higher percentage of body fat (t(53) = 2.90, p = 0.005, Cohen’s d = 0.81). Brain and spine cancer survivors did not have a significantly different body fat percentage compared to survivors with other diagnoses (t(53) = 1.29, p = 0.202, Cohen’s d = 0.41).

**Table 3:** The linear regression model for body fat percentage.

Variable	Level	beta		95% CI	p
diagnosis	Other solid tumours	(ref)			
	Lymphoma	-1.62	.	(-7.58 ; 4.34)	0.587
	Leukaemia	5.46	.	(-4.68 ; 15.60)	0.283
	Brain and spinal column tumours	12.40	.	(0.59 ; 24.21)	0. 0.202
CNS-directed treatment	no	(ref)			
	yes	-6.60	.	(-16.58 ; 3.38)	0.189
sex	males	(ref)			
	females	6.37	.	(1.82 ; 10.93)	0.007
partnership status	no	(ref)			
	yes	5.65	.	(0.67 ; 10.62)	0.027
unemployment	yes	(ref)			
	no	-0.62	.	(-5.74 ; 4.51)	0.809
event free survival (y)	-	0.11	.	(-0.37 ; 0.60)	0.648
age (y)	-	-0.05	.	(-0.65 ; 0.56)	0.873

Table 3 shows the results of linear regression model for body fat percentage. Two of the 55 recruited subjects were removed due to missing data. Multiple R^2^ = 0.37, Adj. R^2^ = 0.24; F(9,43) = 2.85; p = 0.010.

## Discussion

In this study, we investigated the association between selected parameters of socioeconomic status, type of cancer, CNS-directed treatment, and total fat percentage in the Central-European population of childhood cancer survivors. A linear regression model was fitted to explore the association between body fat percentage and cancer diagnosis. Our results show that survivors after brain and spinal column tumours did not have a significantly different body fat percentage compared to survivors with other diagnoses (t(53) = 1.29, p = 0.202, Cohen’s d = 0.41) and women had significantly higher fat levels than men (female vs male: b = 6.37, 95% CI: 1.82–10.93, p = 0.007).

An interesting result of our study was the finding the survivors reporting having a partner had a significantly higher percentage of body fat (t(53) = 2.90, p = 0.005, Cohen’s d = 0.81). Therefore, partnership status may be a potential factor influencing adiposity in this group of survivors. Available studies [5, 31] suggest that childhood cancer survivors are at higher risk of obesity, with studies reporting increased body mass index (BMI) z-scores and a higher prevalence of overweight and obesity in this population. Given their susceptibility to certain health problems, the high prevalence of overweight and obesity observed in survivors of childhood cancer, although similar to the general population, is concerning. Excess body fat and central obesity in childhood cancer survivors are risk factors for cardiovascular disease, insulin resistance, compromised pulmonary function, musculoskeletal dysfunction, altered gonadal hormone levels, and psychological compromise [11]. Although exposures to treatment alone or in combination also contribute to increased cardiometabolic risk in childhood cancer survivors, it is important to note that the proportion attributable to risk found in adult survivors of childhood cancer was <50% and ranged from 9.3% for hypertension to 15.5% for dyslipidemia, 41.7% for diabetes, and 42.1% for obesity [32].

In our study, body fat percentage was measured by bioelectrical impedance analysis (BIA).

In contrast, the body fat percentage values officially reported by the OMA were derived from dual-energy X-ray absorptiometry (DXA) measurements. Thus, our results may be influenced by the difference in measurements of these two methods. An example of the difference in measurement by these two devices can be seen in the study by Achamrah, at el. [33] where the results of the two methods were compared on a population of 653 men and 3002 women. The study showed that regardless of BMI, the BIA and DXA methods showed higher FM values than FFM. BIA and DXA are interchangeable at the population level but agreement at the individual level is lacking.

Furthermore, a significant relationship was found between the CNS-directed treatment and employment status (c^2^(1) = 7.53, p = 0.006, Cramér’s V = 0.38). This effect may be related to intensive treatment. The socioeconomic factors in childhood cancer survivors can significantly impact their long-term outcomes, including educational attainment and psychosocial wellbeing [34]. Erdmann et al. [34] report that childhood cancer survivors, especially those treated for brain and CNS cancers, face a higher risk of obesity and socioeconomic problems. The surgical resection of a CNS tumour or cranial irradiation can lead to irreversible damage to healthy, developing brain tissue and can result in CNS dysfunction and cognitive impairment [35]. Findings from the study by Frederiksen et al. [35] indicate that CCS who are fit to work from a general health perspective have the same employment levels as the general population and as their siblings.

According to a recent review [21], CCS are at a considerable risk of unemployment in adulthood. CNS tumour survivors were almost five times more likely unemployed than controls. This result correlates well with the result of our study, where the highest unemployment rate was found specifically for brain and spinal column tumour survivors (72.7%). compared to other diagnosis groups. Unemployment rates across cancer types were examined in a study [35] which found that the odds of health-related unemployment at age 30 were much higher for CNS cancer survivors than for the comparison population. Significant odds of health-related unemployment were also observed among neuroblastoma and bone tumour survivors, although effect estimates were imprecise due to small samples of these diagnostic groups. In addition, unemployment was high among cancer survivors diagnosed at age <15. According to Wengenroth et al., another potential factor influencing unemployment may be gender. They concluded in their studies that female survivors faced greater health-related barriers to employment [36, 37]. A possible explanation may be that female survivors may be more discriminated against at work or have other life priorities, such as childcare.

Although this study presents a specific group of childhood cancer survivors the clinic for long-term follow-up of childhood cancer survivors, we are aware of certain limitations. Although we reviewed 55 cancer survivors followed up at the clinic, we did not have adequate numbers of all types of cancer, treatment, and other variables to draw definitive conclusions. Future research is warranted to explore the suggested correlations further, including associations with treatment and varying times from diagnosis, to obtain a richer and more accurate picture of the factors determining unemployment in this population and provide further insight into how adverse consequences of cancer and its treatment may be prevented. In our study, body fat percentage was measured by BIA, whereas the body fat percentage values officially reported by the OMA were derived from measurements by DXA. Thus, our results may be influenced by the difference in the measurements of these two different methods.

BIA is a commonly used method for assessing body composition in clinical practice and research studies. BIA is easy to use, non-invasive and inexpensive. It works on the principle of estimating total body water (TBW) through the body’s resistance to a small alternating current [38]. Nowadays, devices are available on the market that work on the multi-frequency BIA (MF-BIA) method, which allows the prediction of intracellular and extracellular water independently of each other and of the phase angle. The phase angle decreases with age and height and increases with greater fat free mass (FFM) in both men and women [39, 40].

Limitations of the BIA method include assumptions involving fixed hydration. These limitations also apply to patients with severe obesity because the distribution of water in the body may be different in severe obesity [41]. Fat mass (FM) is underestimated in this group of patients when measured by BIA [42, 43].

In contrast, DXA is a special imaging method that allows whole-body imaging to measure whole-body bone mass and soft tissue composition. This method works on the principle of two attenuations of X-rays passing through the body, which can be used to accurately calculate the mass of two different materials, assuming simple algebra and the physical properties of these materials [44, 45]. DXA allows you to measure regional body composition by dividing the body using specific well-defined sections, this method can also detect changes in bone density, which is important for diagnosing and monitoring osteoporosis [46]. In addition, DXA is not affected by factors such as hydration levels, food intake or exercise, making it more reliable than BIA [33, 41, 47].

DXA can be considered as a reference method in clinical research because it allows rapid and non-invasive assessment of fat mass (FM), fat-free mass (FFM) and bone mineral density, but its disadvantages are high cost, the need for specialized radiological equipment and poorer feasibility in practice [33]. Last but not least, the DXA examination is associated with ionizing radiation to which the patient is exposed. Even though the effective radiation dose from a single whole-body DXA scan is reported to be < 10 microsieverts [48, 49].

Considering the previous radiation burden of this specific group of patients and the feasibility of measurement in practice and affordability, we chose the BIA method for our study.

The Swiss study [50] concluded that overweight prevalence and risk factors are similar in long-term CCS and their peers. The methods chosen to prevent overweight and obesity in this cohort may be similar to those in the general population. However, the exceptions are CCS treated with cranial radiotherapy and survivors with brain and spinal tumours who may need increased attention during follow-up care.

According to a Nordic register-based cohort study from the SALiCCS research program, survivors at risk of health-related unemployment should be offered comprehensive survivorship care and interventions for obtaining and maintaining suitable employment [35]. The study [51] reported that the issue of "return to work" is a significant problem for long-term survivors of working age. This is because the financial existence of the survivor and their family may deteriorate significantly. Frederiksen et al. points out in their study [35] that cancer survivors classify financial worries about the quality of life as more significant than the physical or psychological side effects of cancer or its treatment.

Excess body fat and central obesity are risk factors for cardiovascular disease, insulin resistance, compromised pulmonary function, musculoskeletal dysfunction, altered gonadal hormone levels, and psychological compromise [5, 52]. All these comorbidities worsen the total condition and quality of life of childhood cancer survivors and may, to some extent, affect their employment. The study results support an income loss hypothesis, indicating unemployment and a higher body fat percentage. The study’s authors from St. Jude [16] examined the effects of socioeconomic status on obesity in survivors. Comparing obese to non-obese survivors found that survivors who lived in a rural area and had lower socioeconomic status were at an increased risk of having higher percent body fat.

There is a strong emphasis on massively supporting intervention programs to promote good nutrition and physical activity to reduce adiposity in patients after cancer treatment [53–56]. Some programs focus on interventions to minimize later mental health disorders and adverse social and socioeconomic consequences of cancer and its treatment [34, 35]. The results of our study show that socioeconomic problems and increased body fat can occur together in survivors with certain diagnoses. By understanding the socioeconomic and health challenges faced by survivors of childhood cancer, tailored intervention strategies can be developed to improve their overall well-being and reduce the long-term impact of cancer and its treatment, including promoting healthy eating and physical activity to reduce the risk of obesity and its associated complications [34, 57]. It is essential to consider the long-term consequences of childhood cancer and its treatment on survivors’ quality of life, including the prevalence of metabolic syndrome, diabetes, and excess cardiovascular morbidity associated with obesity [9]. Interventions to address obesity should be introduced early in the course of treatment, as waiting until survivorship is achieved may be less effective [58].

Interventions for those treated for brain cancer/CNS should target psychological symptoms, health behaviours and socioeconomic outcomes to reduce the risk of obesity and promote overall wellbeing [22]. In addition, interventions should take into account the increased risk of unemployment and lower employment status, particularly for survivors of central nervous system cancers, highlighting the need for targeted support in this subgroup [35, 36]. Cognitive and behavioural interventions have demonstrated efficacy in treating psychological symptoms and modifying health behaviours in childhood cancer survivors [22]. In addition, e-health and mHealth interventions have demonstrated efficacy in targeting emotional distress, health behaviours, and health outcomes in childhood cancer patients, suggesting their potential applicability to childhood cancer survivors [59].

All of the aforementioned intervention options should be tailored to the unique needs of childhood cancer survivors, taking into account their medical history, socioeconomic status and psychological issues, and current health status to promote long-term well-being and reduce the impact of cancer and its treatment [60, 61].

## Conclusion

This study provides a unique insight into a group of adult survivors of childhood and adolescent cancer attending a Brno aftercare clinic. The results of our study suggest that employment in this specific population depends on the type of cancer and the type of treatment, which may also influence the risk of adiposity. In addition, other factors such as realationship status may also contribute to adiposity. Our results could be taken into account in intervention projects and support programs at the national level aimed at this group of survivors, which use modern technologies aimed at obesity prevention and could be very beneficial in promoting the integration of patients into the labour market.
